# How effective is fine motor training in children with ADHD? A scoping review

**DOI:** 10.1186/s12887-021-02916-5

**Published:** 2021-11-04

**Authors:** Miriam Lelong, Annina Zysset, Mirjam Nievergelt, Reto Luder, Ulrich Götz, Christina Schulze, Frank Wieber

**Affiliations:** 1 University of Konstanz, Konstanz, Germany; 2 School of Health Professions, Institute of Health Science, Zurich University of Applied Sciences ZHAW, Winterthur, Switzerland; 3 Zurich University of Teacher Education, Centre for Inclusion and Health in Schools, Zuerich, Switzerland; 4 Zurich University of the Arts, Institute for Design Research, Zuerich, Switzerland; 5 School of Health Professions, Institute of Occupational Therapy, Zurich University of Applied Sciences ZHAW, Winterthur, Switzerland; 6 Department of Psychology, University of Konstanz, Konstanz, Germany

**Keywords:** ADHD, Children, Fine motor skills, Handwriting, Graphomotor skills, Training, Intervention, mHealth

## Abstract

**Background:**

Motor deficiencies are observed in a large number of children with *ADHD*. Especially fine motor impairments can lead to academic underachievement, low self-esteem and frustration in affected children. Despite these far-reaching consequences, fine motor deficiencies have remained widely undertreated in the ADHD population. The aim of this review was to systematically map the evidence on existing training programs for remediating fine motor impairments in children with ADHD and to assess their effectiveness.

**Methods:**

The scoping review followed the *PRISMA-ScR* guidelines. In March 2020, *PsycINFO*, *MEDLINE (PubMed)*, *Web of Science*, *Google Scholar* and *The Cochrane Database of Systematic Reviews* were searched for evidence. The eligibility criteria and the data charting process followed the *PICO* framework, complemented by study design. The investigated population included children with a formal ADHD diagnosis (either subtype) or elevated ADHD symptoms aged between 4 and 12 years, both on and off medication. All training interventions aiming at improving fine motor skills, having a fine motor component or fine motor improvements as a secondary outcome were assessed for eligibility; no comparators were specified.

**Results:**

Twelve articles were included in the final report, comprising observational and experimental studies as well as a review. Both offline and online or virtual training interventions were reported, often accompanied by physical activity and supplemented by training sessions at home. The training programs varied in length and intensity, but generally comprised several weeks and single or multiple training sessions per week. All interventions including more than one session were effective in the treatment of fine motor deficiencies in children with ADHD and had a wide range of additional positive outcomes. The effects could be maintained at follow-up.

**Conclusions:**

Fine motor training in children with ADHD can be very effective and multiple approaches including specific fine motor and cognitive training components, some kind of physical activity, feedback mechanisms, or multimodal treatments can be successful. Training programs need to be tailored to the specific characteristics of the ADHD population. A *mHealth* approach using *serious games* could be promising in this context due to its strong motivational components.

**Supplementary Information:**

The online version contains supplementary material available at 10.1186/s12887-021-02916-5.

## Background

*Attention deficit hyperactivity disorder (ADHD)* is one of the most commonly diagnosed neurodevelopmental disorders with a prevalence ranging from 5 to 7% worldwide [1–3] and a lifetime prevalence of 4.8% in Germany [4]. The condition is characterized by three main symptoms: inattention, impulsivity and hyperactivity - each of which can be predominant or combined in one individual [5]. Although not yet a diagnostic criterion, motor deficiencies are very common in children with ADHD, occurring in 30% up to more than 50% of the individuals [6–9]. Despite the severe impact these motor impairments can have on the daily living, academic achievement and self-esteem of children with ADHD [7, 9–11], they have received little attention and remained widely undertreated so far [7]. Previous treatment approaches focus on the main symptoms, but usually ignore fine motor difficulties. The aim of the current review was to assess the effects different training programs can have on the fine motor skills of children with ADHD.

### Motor deficiencies in ADHD

The prevalence of motor problems in children with ADHD ranges from 30 to 52%, depending on the method of measurement [12, 13]. Pitcher et al. [14] found that the majority of children with ADHD had motor problems.

Numerous studies report a wide range of motor deficiencies in children with ADHD, including reduced handwriting skills, motor control and motor coordination as well as poorer motor programming and movement accuracy [6, 9, 15–19], with movements often being described as jerky or less fluent [15, 20]. Movement speed and temporal organization also seem to be affected [8, 15, 19, 21], although not all studies agree on this point, sometimes attributing the observed differences to an increased movement variability found in children with ADHD [6, 19]. Additionally, they display impairments in balance, body schema, and spatial organization [8, 22]. In general, motor development seems to be anomalous in children with ADHD with a delay of nearly two years compared to neurotypical peers [8, 17, 21, 22].

Deficits are observed both in gross and fine motor skills [9, 16, 18, 20, 23, 24]. However, gross and fine motor performance seem to differ on their underlying behavioral processes [24] and gross motor skills still appear to be a relative strength in children with ADHD compared to their fine motor skills [25], so the focus of this review will be on the latter.

### Fine motor deficiencies in children with ADHD

Many studies show a strong association between ADHD and fine motor problems [14, 24]. Motor problems and fine motor problems lead to difficulties in daily life, including academic performance, sports, play, and self-esteem [26–29]. Motor problems including fine motor problems have a strong impact on children’s daily lives and serve as a predictor of a child’s popularity and self-esteem [30]. These difficulties can have a significant influence on children’s development, leading to difficulties with communication, inhibited social interaction, and poor performance in athletic activities.

According to a *Medline* definition, *fine motor control* involves “the coordination of muscles, bones, and nerves to produce small, exact movements” (*Fine motor control*, 2020) and is “often expressed as handwriting difficulties” [23]. Several studies report that motor deficits in children with ADHD affect all fine motor domains [8, 22, 31, 32] and therefore have an impact on a wide range of life skills as cutting, doing handcrafts or drawing, to name just a few [33].

According to Doyle et al. [25], handwriting appears to be the most dysfunctional motor domain in children with ADHD. As one of the most important daily living activities involving fine motor skills, handwriting will be discussed separately in the following section.

### Handwriting and graphomotor skills in children with ADHD

Deficits in handwriting are very common in children with ADHD. In primary school, children spend most of their cognitive energy managing the spelling and graphomotor aspects of writing [34]. According to estimates by Guinet and Kandel, at least 50% of a child’s school day is spent in writing tasks [35]. Often children diagnosed with ADHD already have difficulties fitting into existing structures and the unwritten rules of everyday school life and additional exercises, like writing, thus are a challenge on multiple levels. In writing, the process and the product seem to be affected [36]. The written material is often described as illegible, inaccurate or inefficient, a phenomenon known as *dysgraphia* [10, 31, 37–41]. The writing size is described as inconsistent or disproportionate [23, 37, 42] and sometimes increasing letter sizes are reported, a phenomenon also referred to as *megalographia* [43]. *Graphemic buffer errors* as letter insertions or omissions are often observed [37, 39].

The handwriting process is characterized by a higher pen pressure [37, 44] which often leads to stiffness and pain in the hand or rapid tiring during writing [37]. Some studies also observed larger variations in pen pressure in children with ADHD compared to neurotypical children [38]. According to some studies, children with ADHD spend more time when writing or make slower strokes than their typically developing peers [36, 37, 39, 41, 44] but the findings are inconsistent. Some studies report faster and more fluent writing or strokes [23], a comparable writing speed to neurotypical children but larger variations or an inappropriate handwriting speed [10, 11, 38].

### Correlates of fine motor impairments in ADHD

#### Subtype

Motor problems seem to be present in all ADHD subtypes although the study situation is very heterogeneous on this topic. According to a review conducted by Kaiser et al. [9], fine motor deficiencies appear to be more present in the inattentive subtype, a finding also reported by Piek et al. [18]. Tseng et al. [24] found inattention and impulsivity to be predictors of both gross and fine motor skills, whereas hyperactivity only seemed to predict gross motor skills. Marcotte and Stern [45] observed graphomotor deficiencies in all ADHD subtypes but the reported impairments were most pronounced in the hyperactive subtype. Meyer and Sagvolden [46] also found motor impairments in all three subtypes but they reported the strongest motor control problems for the ADHD combined type. Piek et al. [18] described more difficulties with gross motor skills in the combined type. According to Brossard-Racine et al. [47], children of all subtypes exhibit handwriting difficulties to a comparable degree, a finding that is in line with Noda et al. [48] who observed reduced handwriting fluency among all subtypes.

#### Gender

Gender does not seem to play a role in the fine motor deficiencies of children with ADHD since both genders appear to be equally affected [6, 16, 46]. The only difference was found for numeral legibility where girls show a better performance than boys [47].

#### Age

In general, motor and handwriting impairments are reported to decrease with age but they still remain prevalent in an important subset of adolescents and adults with ADHD [6, 16, 47, 49]. *Graphomotor learning* in adults with ADHD still seems to be slower than in typically developing controls [50] and *locomotor hyperactivity* stays a characteristic in adult ADHD [51]. According to Meyer and Sagvolden [46], motor control deficiencies are most predominant in children between 6 to 9 years and seem to be attenuated in older children.

#### Ethnicity and culture

General or fine motor impairments are reported across countries in Australian [18, 25], Iranian [33], South African [46], Dutch [16, 52] Brazilian [8, 22] and Taiwanese children [24], to name just a few examples. Handwriting impairments are observed independently of the written or spoken language in English, Chinese [38, 41], Hebrew [36, 53] and Japanese handwriting [48]. Poorer handwriting legibility and speed were observed both in Anglophone and Francophone Canadian children with the latter showing greater speed only in one handwriting subtest [47]. In conclusion, there seems to be no link between ethnicity and handwriting or fine motor impairments [46].

#### Handedness

Meyer and Sagvolden [46] found no influence of handedness, with motor control deficiencies being observed both in the dominant and non-dominant hand.

Based on these findings, the population of the present scoping review was defined. All ADHD subtypes, genders, ethnicities and both right and left handed children were included. The only chosen constraint was the age of the investigated population. The focus of this review was set on school-age children with ADHD, since motor impairments appear to be most pronounced in this age range as stated above.

### Underlying factors of fine motor deficiencies in ADHD

In order to identify possible starting points for fine motor interventions, it is useful to look at the underlying factors of the impairment. Fine motor deficiencies are often linked to abnormalities in the brains of individuals with ADHD. Different hypotheses are proposed for explaining motor impairments in the ADHD population, including the *cortical activation dysregulation hypothesis*, the *cerebellar dysfunction hypothesis*, and the *delayed white matter maturation hypothesis* [8, 54]. Even if the question has not yet been conclusively clarified, numerous studies indicate abnormalities and neurochemical imbalances in brain regions related to motor functions, executive and motor control in individuals with ADHD, including the cerebellum, the premotor cortex, the prefrontal cortex and basal ganglia [6, 8, 23, 43, 55]. Children with ADHD also seem to exhibit a delayed maturation of *transcallosal inhibition*, possibly interfering with the acquisition of fine motor skills [32].

Cerebellar dysfunctions are associated with an increased *intraindividual variability* producing *dysrhythmia* [56], dysmetria [23] and impaired *executive control* [6, 8] all of which appear to be related to deficits in motor control and motor coordination [42, 43]. Visual motor integration and upper extremity coordination predict handwriting legibility [54] and children with ADHD show a poorer performance in these domains than their typically developing peers [41]. Motor programming seems to be impaired in individuals with ADHD [15, 19], also affecting motor control. Seli et al. [57] found mind-wandering to interfere with task-related executive control and therefore to be an underlying factor of motor control deficits in the ADHD population. Schoemaker et al. [44] reported a deficiency in parameter setting in affected individuals, a motor component also related to executive functions and therefore to *response inhibition*.

In sum, reduced executive control seems to present one of the main underlying factors of fine motor impairments in children with ADHD [21] and leads to a decreased behavioral inhibition [8]. The *stimulation deficit hypothesis* provides an additional explanation for motor abnormalities in children with ADHD [58–60].

### How the intervention might work

Lipowska [39] questioned whether graphomotor impairments were attributed to actual fine motor deficiencies or if they only presented a side effect of a planning deficit. She hypothesized that graphomotor problems could be related to both underlying causes. These findings are consistent with Feder and Majnemer [61] who concluded that poor handwriting and fine motor outcomes could either be a product of actual motor impairments or they could occur due to external environmental factors. Therefore, three starting points for possible fine motor interventions seem plausible:

1. Interventions targeting fine motor skills directly
2. Interventions aiming at improving ADHD symptoms and thereby having an indirect effect on fine motor skills
3. Interventions altering situational or environmental factors enabling a better fine motor performance

The first type of intervention will be the main focus of this review. Since ADHD symptoms appear to play a role in fine motor difficulties of children diagnosed with the disorder [31], the second type of intervention will also be included in the present review. As seen before, different fine motor domains are related to the ADHD subtypes or main symptoms and symptom severity seems to predict the degree of impairment [11, 16, 18, 23, 24, 31, 48]. In addition, the execution of movements, especially in the fine motor domain, requires increased attention [25]. Dahan et al. [20] proposed a model of the motor regulation process comprising four stages: “attention to target, motion preparation, motion execution, and motion monitoring” (p. 34) with attention being involved in all stages and therefore being crucial for the successful execution of a desired movement. This leads to the assumption that a training intervention targeting ADHD inattention could also lead to an improved fine motor performance.

The third type of intervention does not involve training programs and will therefore not be covered by this review but a brief outline on related findings will be given in this section. External factors as medication or environmental factors as stimulation can be altered in order to improve fine motor skills in children with ADHD. A wide range of studies support an underlying stimulation deficit in individuals with ADHD that in part can account for the observed symptoms [58–60, 62, 63]. Stimulations as colored paper, reduced classroom noise, classroom seating on therapy balls or the use of weighted vests yielded improved handwriting outcomes in ADHD subjects [6, 58, 60, 63–66]. The evidence on drug treatment will be summarized in the subsequent section.

### Why it is important to do this review

#### The need for ADHD-specific, non-pharmacological interventions

“Where the basis of motor problems is deemed to be related to the signs of ADHD, it is unlikely that the usual occupational therapy [programs] for motor skills difficulties will be most effective” [25]. According to this statement by Doyle et al. [25], the following section aims to clarify why there is a strong need for ADHD-specific interventions.

As mentioned earlier, individuals with ADHD show specific neuroanatomical, neurological and developmental characteristics which distinguish them from their typically developing peers. The brain characteristics of ADHD subjects require specific interventions tailored to their individual weaknesses and needs. Individuals with ADHD also show differences in motor learning, exhibiting impaired graphomotor procedural learning [50] as well as differences in motion execution [19]. For example, arm movements are not performed as a functional unit as do neurotypical children and children with ADHD seem to rely on visual feedback to correct their movements. Since the whole process of movement execution appears to differ from typically developing peers, children with ADHD need a different approach for remediating problems in this area. Another argument for ADHD-specific fine motor trainings is the observed delay in the motor development of children with ADHD compared to neurotypical children [8, 21]. These findings emphasize the importance of fine motor interventions at an early age in order to prevent the consolidation of motor deficits [21]. In sum, a “one size fits all” approach without taking into account the individual characteristics of the ADHD population does not seem appropriate when the underlying factors are ignored.

The need for non-pharmacological interventions becomes evident when looking at the evidence of drug treatment for fine motor impairments in children with ADHD. Several studies found persisting handwriting or fine motor deficiencies in a considerable proportion of patients treated with stimulant medication [9, 67, 68]. In addition, drug treatment was reported to even reduce handwriting fluency [49, 52]. Some studies found placebo to be equally efficient as methylphenidate in remediating motor impairments, thus showing no advantage of the latter [68, 69]. Brossard-Racine et al. [67] concluded that medication alone was not a sufficient solution for the treatment of fine motor deficiencies. Maier [70] suggested a multimodal treatment approach for ADHD, combining stimulant medication with behavioral or cognitive interventions.

### Implications of fine motor impairments

The importance of treating fine motor deficiencies becomes apparent when considering the severe impact these impairments can have on the lives of children with ADHD when remaining untreated [7]. Especially handwriting consists of an affected life skill that can have far-reaching negative consequences, including lower academic achievements and a lower self-esteem [10, 11, 40, 61]. Children with dysgraphia seem to systematically stay below their intellectual potential in all academic areas, especially when writing difficulties are combined with impaired attention [11]. Handwriting difficulties can lead to reduced participation in daily living activities [9], frustration and writing avoidance [71], homework stress and dislike of school [40]. To break out of the vicious cycle of avoidance, lacking writing practice and negative feedback, the development of self-efficacy for writing seems crucial [72].

In conclusion, there is a clear need for ADHD-specific, non-pharmacological interventions to break the negative cycle of fine motor impairments in children with ADHD. Despite the striking evidence of motor impairments in children with ADHD, these have remained widely undertreated [7]. The aim of this review is to explore and summarize the existing research on fine motor trainings of children with ADHD, to clarify if and how they can be successful and to identify core elements that have proven to be effective in the treatment of fine motor impairments in ADHD.

## Objectives

A scoping review was conducted in order to systematically map the existing evidence on the effectiveness of fine motor skills training in children with ADHD and to consequently provide a starting point for future research and the development of effective training programs. The review was guided by the following research question: How effective is fine motor training in children with ADHD?

## Methods

### Study design and protocol

A scoping review was conducted to address the present research objectives. The *PRISMA Extension for Scoping Reviews* (*PRISMA-ScR* [73];) served as a guideline for reporting. A protocol does not exist, as this would have gone beyond the scope of a student thesis.

### Eligibility criteria

The inclusion criteria were defined according to the *PICO framework* (Population, Intervention, Comparator, Outcome [74, 75];) and complemented by study design. Other characteristics as language and research area were used as additional criteria.

#### Population

The population of interest consisted of children aged between 4 and 12 years with a formal *ICD-10* or *DSM-5* ADHD diagnosis [5, 76] or elevated ADHD symptoms. The age range was chosen to represent preschool and school children. All ADHD subtypes and both children on and off medication were eligible for inclusion.

#### Intervention

Training interventions aiming at improving motor functions or performance, having a motor component or having an impact on motor performance were eligible. The search was not further limited to fine motor skills, as there are only a few studies on fine motor training available to date. In addition, studies investigating motor skills often include fine motor skills as one of the principal motor components, so broader eligibility criteria were justified. Trainings that led to improved handwriting were also considered for inclusion.

#### Comparator

Any comparator was relevant for inclusion as well as studies without comparators.

#### Outcome

Studies on fine motor skills, handwriting skills, *graphomotor skills*, *visuo-motor skills*, *dexterity* and other fine motor associated outcomes as measured by standardized tests (e.g. *Bruininks-Oseretsky Test of Motor Proficiency (BOTMP)*; *Beery-Buktenica Developmental Test of Visual-Motor Integration (BEERY VMI)*; *Canadian Occupational Performance Measure (COPM)*), parent, teacher or self reports, specific graphomotor softwares or other tests were relevant for the present review.

#### Study design

All study designs and publication types were eligible for inclusion because of the narrative nature of this review.

#### Other

Publications written in English, German, French or Spanish were eligible for inclusion. For one database, the search was narrowed down to English articles, school aged children (6–12 years) and human population at the beginning. However, these limiters seemed to be too specific, so only the age constraint was maintained for a second search. On a second database, the search was refined by specific research areas related to health care, psychology, neurology, rehabilitation and similar domains to yield a more specific result.

### Information sources

#### Electronic searches

The following databases and web search engines were searched in March 2020 in the presented order.

1. PsycINFO
2. Web of Science
3. MEDLINE (PubMed)
4. Google Scholar
5. The Cochrane Database of Systematic Reviews

Since the research topic of the present scoping review lies at the interface between psychology, medicine, occupational therapy and gaming, the aim of the database selection was to cover a broad spectrum of articles from a wide range of research areas. PsychINFO was searched for psychological literature while MEDLINE (PubMed) was selected to cover medical evidence. Web of Science and Google Scholar were used for a more sensitive search including different research areas. No specific databases were found for gaming literature or occupational therapy studies so additional resources were handsearched as reported below.

#### Searching other resources

The electronic database search was supplemented by handsearching the following websites in March 2020.

1. Schreibmotorik Institut (https://www.schreibmotorik-institut.com/index.php/de/publikationen)
2. Amy Lu (https://web.northeastern.edu/amylu/publications.html)

The first website with its focus on writing motor skills was chosen for the investigation of occupational therapy literature. The second website offers an overview of Amy Lu′s publications, whose research on gaming often includes a health or therapeutic perspective. Further studies were selected from a private collection of thematically related literature that was created in 2018 for other academic purposes. Literature suggestions of the supervising professor were also included. The literature search was extended by scanning of reference lists of relevant articles and reviews in August 2020 and by handsearching a private literature collection of the research team on *Zotero*. One last study was identified in September 2020 through snowballing. To prevent risk of bias, grey literature was also included in the research. For this purpose, archived Bachelor and Master theses were requested from the university. Additionally, a fellow student was contacted and asked for her Bachelor thesis on a related topic. Lastly, some grey literature was provided by the supervising professor.

### Search strategy

The search strategy was developed according to tips and guidelines from educational material provided by the supervisor and requested from a second instructor. Since this is a student thesis, the search strategy was not peer-reviewed. *MeSH* terms were developed following key concepts of the research question and refined by generating synonyms and related terms. The final thesaurus is reported in Additional file 1: Appendix 1 (in Supplementary) but was not used for the literature search since the results yielded in a test trial were not specific enough. Instead, search strings were generated using truncation, phrase searching and Boolean operators to link the different MeSH terms, to narrow or broaden the search (*see* Additional file 1: Appendix 2). The complete search strings for the three main databases are available in Additional file 1: Appendix 3. Limitations and filters applied for narrowing down the search results are also reported in Additional file 1: Appendix 3 and the rationale is provided in the section about eligibility criteria. Grey literature was obtained through a request and provided by the supervising professor as mentioned above. According to the university librarians, a collection of former theses no longer exists, so a systematic grey literature search could not be conducted as planned.

### Selection of sources of evidence

A multi-level process of selecting sources of evidence following the *PRISMA Statement* [77] was conducted. The whole screening process along with the reasons for exclusion was documented in a spreadsheet (obtainable through author request) but no standardized form or software was used for article selection. A calibration exercise could not be conducted for this student thesis as this would have required a team of at least two reviewers to test agreement on study selection or inter-rater discrepancies.

**Fig. 1:**
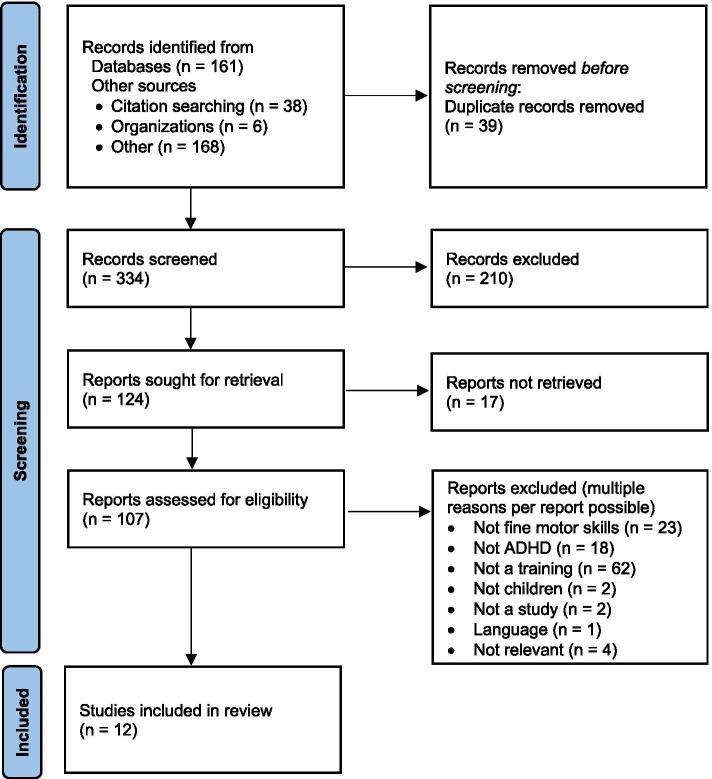
PRISMA Flow Diagram

In a first step, the titles and abstracts of records identified by the search were screened. A rather sensitive approach was chosen due to the very limited number of studies on the topic. Only articles that clearly did not meet the predefined eligibility criteria were excluded at this first stage of screening. In case of doubt, the full text was retrieved for a second stage screening. For the articles considered appropriate for this review, the full text version was also obtained. If the text was unobtainable or the access was denied, the authors were contacted for providing their research. Each full text article was reviewed following the eligibility criteria. Duplicates were screened by arranging the study titles documented in the spreadsheet in an alphabetical order. The results of the screening process were recorded in a *PRISMA flow diagram* (*see* Fig. 1 [77];).

### Data charting process and data items

The data charting process was conducted independently. A data-charting form was prepared according to the PICO reporting system [74, 75] and discussed with the supervisor who approved the planned outline. Data were extracted on study design, population (sample size, age, diagnosis, medication, control groups), intervention (description, treatment schedule, comparator, additional notes), outcomes (including outcome measures for operationalization). No additional software or calibration was applied in the process. Table 1 presents the final version of the charting form.

**Table 1:** Summary of Included Sources

Source	Study design	Population	Intervention	Outcomes (incl. outcome measures)
Bartscherer & Dole (2005) [78]	Case report	Boy with attention and motor coordination difficulties (*n* = 1; no formal ADHD diagnosis) **Age:** 9 **Medication:** no	**Interactive Metronome training:** Variety of upper and lower limb tasks performed with metronome beat **Schedule:** 7-week program Sessions 3x/week (60–90 min each) 4 pre-sessions, 15 sessions	**Bruininks-Oseretsky Test of Motor Proficiency (BOTMP):** Improvements in gross and fine motor skills: ∙ Largest improvements in balance, response speed, visual-motor control, upper limb speed, dexterity ∙ Smaller changes in bilateral coordination, strength ∙ No change in running speed, agility ∙ Decline in upper limb coordination **Interactive Metronome long-form test (IM LFT):** Improvements in timing accuracy **Parent reports:** Behavior changes More cooperative, less resistance & fighting, self-confidence (willingness to take risks), faster at solving math problems, improvements in handwriting
Dahan et al. (2018) [20]	Review	ADHD subjects	**Neurofeedback (NF) interventions:** Theta/beta frequency training and SCP neurofeedback **EMG-Biofeedback (EMG-BF) interventions:** Feedback on motor activity **Physical activity and motor interventions:** Several weeks training program or single intervention (e.g. running on treadmill, ball handling and balance exercises, general exercise)	**NF:** Improvements in behavioral self-regulation; reduction of ADHD symptoms (mixed findings); increase in response speed; no differences between the different NF approaches **EMG-BF:** Improvements in motor coordination and regulation, visuo-motor precision, flexibility, fine motor control, muscle relaxation; reduction of ADHD symptoms **Physical activity and motor interventions:** Improvements in executive functioning, social behavior, response preparation, working memory, motor performance; reduction of ADHD symptoms; regardless of type of physical activity **Follow-up:** Evidence for persisting changes
Duda et al. (2019) [79]	Case-control study	Children and adolescents with ADHD (*n* = 16) **Control group:** Children and adolescents without ADHD (*n* = 16) **Age:** 9–15 **Medication:** no	**Graphomotor learning task:** Practicing a novel grapheme on a digitizing tablet 30 x **Schedule:** One single session **Note:** Not a training for fine motor skills but to investigate graphomotor procedural learning	**WACOM Cintiq 21UX digitizing tablet & MovAlyzeR software** **ADHD group:** No improvements in graphomotor fluency and automaticity **Control group:** Improvement in graphomotor fluency and automaticity
Gharebaghy et al. (2015) [80]	Single case experimental design (multiple baselines)	Children with ADHD (*n* = 6) **Age:** 7–12 **Medication:** yes	**Cognitive Orientation to daily Occupational Performance (CO-OP):** Use of cognitive strategies to reach self-selected goals regarding motor performance in daily living activities (e.g. handwriting, dressing) **Schedule:** 12-week program 12 sessions (45–60 min each)	**Bruininks-Oseretsky Test of Motor Proficiency and Performance (BOTMP):** Improvements in motor performance **Canadian Occupational Performance Measure (COPM):** Perceived improvements in chosen goals (satisfaction and performance) reported by all children and parents **Goal Attainment Scaling:** 17/18 goals attained or exceeded
Halperin et al. (2013) [81]	Proof-of-concept study	Preschool children with ADHD (*n* = 29; all subtypes) **Age:** 4–5 **Medication**: no	**TEAMS Intervention (Training Executive, Attention, and Motor Skills):** Games designed to enhance inhibitory control, working memory, attention, visuo-spatial abilities, planning, and motor skills (e.g. games with balls, puzzles, jump rope) + additional aerobic exercises (e.g. jumping jacks) **Schedule:** Therapist sessions for children (90 min each) Parent sessions (20 min each): Psychoeducation & support **Group A:** 5 weeks, 1x/week **Group B:** 8 weeks, 1x/week **Group C:** 5 weeks, 2x/week **A, B, C:** Additional daily training sessions at home (30–45 min each)	**ADHD-RS-IV (parent & teacher ratings):** Improvement in ADHD symptoms; reductions in impairment No differences on outcomes among groups **Parent Satisfaction Questionnaire (PSQ):** Satisfaction with program and treatment compliance **Follow-up (1 month & 3 months):** Improvements in ADHD symptoms maintained
Molsberger et al. (2014) [82]	Case report	Boy with ADHD (*n* = 1) **Age:** 9 **Medication:** yes	**Complementary medical intervention: Applied kinesiology (AK), acupuncture, respiratory exercises** **Schedule:** 10 months 18 sessions Week 1: 2 sessions Week 2 to end: sessions every 2–4+ weeks First 3 sessions: respiratory exercises (10–15 min each) + acupuncture (permanent needles for 2 days each time) Respiratory exercises at home (2 min/day)	**Coachman’s test:** Normalized muscle function **Parent reports:** Improvements in sleep behavior and handwriting; increased effectiveness of medication after acupuncture **Follow-up (15 months):** Improvements maintained
Palsbo & Hood-Szivek (2012) [83]	Non-randomized uncontrolled pretest-posttest design	Children with learning impairments, neuromotor and/or handwriting deficits (*n* = 18): ASD (*n* = 5), ADD or ADHD (*n* = 2), Pervasive developmental delay (*n* = 1), Intellectual disability (*n* = 2), Auditory processing disorder or deafness (*n* = 2), no disability (*n* = 6) **Age:** 5–11	**Robotic-assisted three-dimensional repetitive motion training:** Use of a haptic computer-user interface to improve handwriting through a program of active therapy (e.g. proprioception exercises, robot-assisted glyph formation) with multisensory feedback **Schedule:** 4–6/8 weeks 15–20 sessions 3-5x/week or daily sessions (25–30 min each)	**Beery-Buktenica Developmental Test of Visual-Motor Integration (Motor Coordination Subtest):** Improvements in fine motor skills & motor control (children with learning disabilities and ≥ 9 years) No improvements (children with CP or < 9 years) **Test of Handwriting Skills-Revised (THS-R; random number and uppercase letter order subtests) & Print Tool (random lowercase letter order subtest):** Improvements in consistency of glyph formation (10/14 children) and writing size (all children with ASD) **Evaluation Tool of Children’s Handwriting (ETCH; copy subtest):** Improvements in handwriting fluidity (writing speed) while maintaining legibility (all children with ASD or ADHD/ADD)
Ruiz-Manrique et al. (2014) [84]	Case report	Boy with ADHD and comorbid video game addiction (*n* = 1) **Age:** 10 **Medication:** yes	**“ADHD Trainer”:** Mobile/tablet application designed to treat ADHD using a cognitive training method to enhance cognitive skills (attention, working memory, processing speed, calculation ability, reasoning, visuo-motor coordination) **Schedule:** 2 months (within study), 6 months (total training reported by parents) 1st month: 10 min-4 h/day (average: 1 h/day) 2nd month: min. 10 min/day	**Conners’ Parent and Teacher Rating Scales & Barkley School Situations Questionnaire:** ∙ Behavioral improvements ∙ Academic improvements ∙ Improvement of cognitive areas: visuo-spatial working memory, fine motor skills **Parent report:** Reduction of videogame abuse (playing time)
Shaffer et al. (2001) [85]	Randomized controlled pretest-posttest design (blinded)	Boys with ADHD (*n* = 56) **Age:** 6–12	**Interactive Metronome training:** Variety of upper and lower limb tasks performed with metronome beat **Schedule:** **Treatment group:** 3–5 weeks Interactive Metronome training 15 sessions (60 min each) **Control group A:** No intervention **Control group B:** 3–5 weeks video game training (incl. eye-hand coordination, advanced mental planning, multiple task sequencing) 15 sessions (60 min each)	**Treatment group:** Improvements in attention: ∙ **Tests of Variables of Attention (TOVA):** Reduction of errors and distractibility, consistency of reaction time, improved overall attention ∙ **Conners’ Rating Scales-Revised (CRS-R) Teacher & Parent versions:** Improvements in aggression control ∙ **Wechsler Intelligence Test for Children-Third Edition** ∙ **Achenbach Child Behavior Checklist** Improvements in motor control: ∙ **Conners’ Rating Scales-Revised (CRS-R) Teacher & Parent versions** ∙ **Achenbach Child Behavior Checklist** ∙ **The Sensory Profile** ∙ **Bruininks-Oseretsky Test for Motor Proficiency (selected subtests)** Improvements in academic achievements: ∙ **Wide Range Achievement Test (WRAT 3; reading & writing):** Improvements in reading ∙ **Language Processing Test:** Improvements in language processing **Differences between groups:** Improved performance in treatment group; decreased performance in control group and video game group
Tucha & Lange (2005) [86]	**Study 1:** Experimental design (randomized) **Study 2:** Experimental design (randomized) **Study 3:** Case report	**Study1:** Neurotypical students (*n* = 26) **Age:** 20–35 **Study 2:** Children with ADHD/combined type (*n* = 12; no fine motor problems) **Age:** 9–12 **Medication:** Depending on experimental condition **Study 3:** Boy with ADHD/combined type (*n* = 1) **Age:** 10 **Medication:** yes	**Study 1:** Examination of handwriting movements of neurotypical students under different conditions (normal/neat/blind writing, visually/mentally tracking the pen) **Schedule:** 1 single session **Note:** Not included in review (not ADHD) **Study 2:** Examination of handwriting movements of children with ADHD under medication vs. placebo condition **Medication condition:** different instructions (repetitive hand movements, fluent circle drawing, blind/fast writing) **Schedule:** 2 sessions in 5–7 days **Note:** Not included in review (no training) **Study 3:** Training of automated handwriting movements (copying short texts) aided by simple instructions to direct attention away from accuracy and legibility of handwriting **Schedule:** ~ 3 weeks 6 sessions	**WACOM IV Digitizing Tablet & specific pen:** **Study 1 (Tablet):** Automated handwriting movements under different conditions **Study 2 (Tablet):** Reduced handwriting fluency on medication; automated handwriting movements on medication through instructions **Study 3:** ∙ **Tablet:** Automated and perfectly smooth handwriting movements (on tablet and homework) through instructions and feedback; reduced number of false starts ∙ **Parent and teacher reports:** Increased writing speed; legible handwriting but irregular alignment; better grades; higher motivation **Follow-up (4 weeks):** Improvements of handwriting movements maintained
Weerdmeester et al. (2016) [87]	Feasibility study (randomized controlled pretest-posttest design)	Children with ADHD (*n* = 47) or elevated ADHD symptoms (*n* = 26) **Age:** 6–13	**“Dragon”:** Full-body videogame intervention with ADHD-focused training components **Intervention condition:** “Dragon” **Control condition:** “Angry Birds Trilogy” (comparable full-body videogame not targeting ADHD symptoms) **Schedule:** 6 sessions (15 min each)	**ADHD VragenLijst (AVL; teacher-ratings):** Greater improvement of ADHD symptoms in intervention vs. control group **Go/no-go task:** Reduction in number of hits (sustained attention) in both groups; greater increase in false alarms (impulsivity) in intervention vs. control group **Movement Assessment Battery for Children (MABC-2-NL):** Improvements in fine motor skills (both groups); no improvement in gross motor skills **Evaluative questions about “Dragon”:** Satisfaction with game
Yazd et al. (2015) [88]	Experimental design (randomized)	Children with ADHD (*n* = 36) **Age:** 6–12 **Medication:** Depending on experimental condition	**Group A:** Perceptual-motor training (incl. spatial/temporal/directional/body awareness, balance, coordination) **Group B:** Combination of perceptual-motor training and drug therapy **Group C:** Drug therapy (Methylphenidate, Risperidone) **Schedule:** 6-week treatment/training **Group B & C:** 18 training sessions	**Bruininks-Oseretsky Test of Motor Proficiency:** ∙ **Group A:** Improvements in gross and fine motor skills ∙ **Group B:** Improvement in gross and fine motor skills ∙ **Group C:** No improvement in motor performance

### Quality appraisal and risk of bias assessment

In line with the PRISMA-ScR guidelines [73], no quality appraisal or risk of bias assessment was conducted since the aim of this scoping review was to systematically map the research done in this area.

### Synthesis of results

The evidence will be presented both in a narrative and in a tabular format. The narrative description of study results includes a summary of the study and population characteristics, the interventions and the outcomes. The types of interventions are clustered in three categories depending on their direct or indirect relation to fine motor skills. A description of the training programs will be provided, followed by a summary of the different treatment schedules. The section on outcomes covers the main findings along with secondary outcomes, possible side effects and long term effects of the interventions. The effects of the different studies will not be compared due to the heterogeneity of the included sources and of their outcome measures.

## Results

### Selection of sources of evidence

#### Identification

The search yielded a total of 373 records, of which 161 were a result of the database search. The first search string identified only 21 records on PsycINFO, so the search string was refined (see Additional file 1: Appendix 3). The second version already resulted in 84 records, including the 21 hits from the first search. For MEDLINE (PubMed), a total of 33 records were identified and the search on Web of Science yielded 112 results in the first run. The identified records were not considered specific enough so they were not counted or screened for titles and abstracts. Instead, the search was refined by some limiters and yielded 44 more specific results in the second run. No relevant records were found on The Cochrane Database of Systematic Reviews whereas Google Scholar, even after narrowing down the search criteria, delivered such a large number of hits that the amount of data could not be handled alone and thus was excluded.

Two hundred twelve additional records were identified through other sources. On *Amy Lu’s website*, 47 publications were available and the website from *Schreibmotorik Institut* provided 75 publications. 38 articles were identified through snowballing and eight articles were provided by the supervisor. Another 18 studies stemmed from a private collection of related literature and 25 articles were found through the literature collection of the research team. Lastly, one article was requested from a fellow student.

#### Screening

After removing 39 duplicates, 334 records remained for the selection process. After title screening, 162 records were excluded because they were not relevant to the objectives of this review. During abstract screening, another 48 irrelevant records could be excluded. Two abstracts were not found, so the studies were also excluded at this stage.

Ninety-nine publications were retrieved whereas 15 articles were not found or the access was denied. Nine articles were requested from the respective authors out of whom eight authors were willing to share their research. One identified study was still ongoing and therefore excluded from this review. In sum, a total of 107 full-text articles were assessed for eligibility.

#### Eligibility

Ninety-five full-text articles were excluded during second stage screening for different reasons. The majority of the excluded articles did not meet the predefined eligibility criteria and some publications even violated several inclusion criteria. For example, a lot of studies provided a descriptive report of fine motor skills in children with ADHD but did not conduct any training to improve these skills. Other studies reported on interventions but not trainings, so they were not relevant for the objectives of this review. Some studies implemented interesting trainings of fine motor skills or handwriting, but the population did not meet the eligibility criteria, either consisting of neurotypical children, of children with other diagnoses or of adults with ADHD. After full text screening, four articles were considered to be irrelevant for the review, one study was excluded because it was written in Turkish language and another two records turned out not to be scientific studies.

Twelve publications met the eligibility criteria and were included in the final synthesis. The whole process of article selection is illustrated in a PRISMA flow diagram (*see* Fig. 1 [77];).

### Characteristics and results of sources of evidence

All of the included sources were published after 2000. The study designs were very heterogeneous, comprising five observational studies (including four case reports), eight experimental studies and one review [20]. One publication reported three studies, so the total count of study designs was 14, but 12 full articles were included as described above. Dahan et al. [20] included two of the studies identified for this thesis [83, 85] in their review but it provided a lot of additional information including a variety of studies that were not found or reported otherwise in this scoping review.

A description of the different interventions aiming at improving fine motor skills or handwriting skills, including a motor component or having (fine) motor improvements as positive side effects is provided in Table 1. It also presents the main outcomes alongside with the outcome measures as an operationalization of the research question. The study designs, the characteristics of the investigated populations and eventual comparators are also described.

### Synthesis of results

#### Population

The 11 studies included a total of 292 participants ranging from 4 to 15 years of age. The total sample size of the studies reviewed by Dahan et al. [20] is not known. Two studies also included adolescents, thus exceeding the set age limit [79, 87], but they were still selected for the final synthesis since the samples also consisted of children meeting the predefined age criterion. One of the three studies reported by Tucha and Lange [86] included a group of neurotypical students aged between 20 and 35 years and was therefore not considered to be relevant for this review.

Two hundred thirty-four children had a formal ADHD diagnosis or elevated ADHD symptoms, 10 subjects had other diagnoses and 48 participants were neurotypical controls. All subtypes were represented among the ADHD subjects as well as different medication statuses.

#### Interventions and comparators

The interventions included in this scoping review can be summarized in three clusters:

##### Trainings aiming at improving fine motor skills or handwriting skills

Two studies implemented *Interactive Metronome training* [78, 85] that consisted of a variety of upper and lower limb tasks performed to a metronome beat. In their review, Dahan et al. [20] also reported on Interactive Metronome training as a possible intervention for improving motor deficiencies in ADHD. A few other general physical activities and more specific motor interventions were also included but the main focus of the review was on *Neurofeedback Interventions (NF)* and *EMG-Biofeedback (EMG-BF)*, both measuring biological parameters and giving feedback on those to improve motor performance. Palsbo and Hood-Szivek [83] provided multisensory feedback in their *robotic-assisted three-dimensional repetitive motion training* of the handwriting and fine motor skills of children with different diagnoses, including ADHD. In their case report, Tucha and Lange [86] also used feedback and other verbal instructions to help generating automated handwriting movements during a writing training of a boy with ADHD.

Some researchers devised specific training programs aiming at improving fine motor skills. For example, Halperin et al. [81] developed the *TEAMS intervention for training executive, attention and motor skills* in children with ADHD through multiple games targeting different problem areas and additional physical activity. In one study [80] children set their own goals regarding motor performance in daily living activities and were assisted in reaching these goals by the *Cognitive Orientation to daily Occupational Performance (CO-OP)* program. Yazd et al. [88] compared the effectiveness of *perceptual-motor training* to drug therapy and to a combined treatment approach for improving gross and fine motor skills in children with ADHD. The perceptual-motor training comprised a variety of exercises aiming to train motor awareness, balance and coordination.

##### Trainings including a motor component

In a case-control study, Duda et al. [79] compared the graphomotor learning process of children with ADHD with a control group of neurotypical children in a simple graphomotor task. It should be noted that this was not a real training, as the examination included only a single session, but the repetition of the graphomotor task could be described as some kind of training. The study was still relevant to the objectives of this review in describing the (lacking) effectiveness of a simple fine motor training in children with ADHD and by comparing the outcomes to a control group to identify specific aspects of graphomotor learning that must be considered in children with ADHD. Weerdmeester et al. [87] developed a full-body videogame intervention with ADHD-focused training components aiming at decreasing ADHD symptoms. The intervention had a strong motor orientation and gross and fine motor skills were assessed as additional outcome measures.

##### Trainings having motor improvements as a positive side effect

In a case report, Molsberger et al. [82] described a complementary medical intervention used for the treatment of a boy with ADHD. The treatment consisted of *applied kinesiology (AK)*, *acupuncture* and respiratory exercises. Although the study did not appear very scientific and could be biased, it was still included in the synthesis to provide an alternative therapeutical approach as an addition to the many scientific approaches reported in this review. Ruiz-Manrique et al. [84] developed the *ADHD Trainer*, a mobile application designed to treat ADHD using a cognitive training method to enhance cognitive skills. Although they were not the main focus of this study, fine motor skills were assessed as a complementary outcome.

#### Frequency and duration of the interventions

The majority of the reported training programs had a total duration of four to eight weeks. The shortest intervention only lasted about three weeks and comprised six sessions in total [86]. One study reported a 12-week training program [80] and another study included an intervention of two months but the training was continued at home so the total duration of the intervention was six months [84]. The longest reported training period comprised a 10-month program [82].

Some of the studies only included trainings guided by the researchers, but other programs were supplemented by individual training sessions at home, often accompanied by the parents. The frequency and intensity of training sessions varied between studies. Some treatments took place on a weekly basis, whereas other interventions involved daily sessions. In the majority of the studies, training sessions were scheduled three times per week. The duration of individual sessions ranged between 15 to 90 min, often lasting about 1 h. One study fixed a minimum of 10 min of daily practice and a maximum of 4 h per day on a mobile application [84]. Halperin et al. [81] compared different training frequencies and intensities in their proof-of-concept study and found no differences between the different conditions involving either five or eight weeks of training with one or two sessions per week.

#### Outcomes

All of the studies included in this review reported an improvement of fine motor skills, handwriting, visuo-motor skills or (fine) motor control after the completion of the training programs. Only one study that did not aim at improving fine motor skills in the first place, reported no improvements by the mere repetition of a graphomotor task in a single session [79]. Only a very small number of participants did not improve in the targeted domains, often attributed to a lack of compliance to the program.

Further positive outcomes could be observed in nearly all of the interventions, including improvements in gross motor skills, self-regulation, executive functioning, timing accuracy, academic achievements or reductions of ADHD symptoms as well as behavior changes like improvements in social behavior, aggression control, sleep behavior or videogame abuse. A lot of participants expressed their satisfaction with the program and treatment compliance was generally high, but the palatability was not assessed in all of the studies.

##### Side effects

No severe side effects were observed although one study reported a decline in upper limb coordination [78] and in another study an irregular alignment of handwriting was observed [86]. Weerdmeester et al. [87] reported a decreased performance on the *go/no-go task* after the intervention, but some of these changes could also be noted in the control group. Overall, the positive outcomes outweighed these side effects by far in all three studies and the trainings proved effective in remediating a range of fine motor functions.

##### Long term effects

Most of the outcomes were reported during the intervention or directly after completion of the training program. Dahan et al. [20] reported some evidence of persisting motor improvements after a follow-up period. Three further studies included a follow-up examination, reporting that improvements in handwriting [82, 86], ADHD symptoms [81], muscle function and sleep behavior [82] could be maintained at follow-up.

## Discussion

### Summary of evidence and implications for practice

The articles included in this scoping review encompassed a wide range of different training interventions for the treatment of fine motor impairments in children with ADHD. Some of the interventions aimed directly at improving fine motor skills or handwriting, whereas other studies had fine motor improvements as a secondary outcome and some treatments involved a fine motor component and were therefore included in this review. Regardless of the type of intervention, all of the included sources reported improvements in fine motor skills or related domains in children with ADHD after completion of the intervention. The only exception was a case-control study [79] that did not involve any training program but rather a single training session where a graphomotor task was repeated several times. The study was still included in the present review because it highlights the necessity of specific training programs and shows that the simple repetition of a fine motor task does not seem to improve performance in children with ADHD as opposed to typically developing peers. This finding is in line with previous research on attenuated graphomotor program learning in adults with ADHD [50].

The reviewed literature provides preliminary evidence for the effectiveness of training programs in improving fine motor skills in children with ADHD. The reported outcomes encompassed improvements in several fine motor domains as handwriting, visuo-motor skills and fine motor control. A wide range of additional positive outcomes were observed, ranging from improved gross motor skills, general motor control and timing accuracy, decreased ADHD symptoms, better self-regulation and improved executive functioning. Higher academic achievements were also eventually reported as well as behavioral improvements. The overall satisfaction and attendance to the training programs was high, both for the ADHD children and for their parents. No severe negative side effects were observed, leading to the conclusion that fine motor interventions can be safely implemented in the ADHD population. All studies including a follow-up reported that the positive effects of the training could be maintained after completion of the program.

The implemented training programs had different intensities regarding the frequency or duration of training sessions and the overall scope of the program. Except the single-session training used by Duda et al. [79], all training programs were effective and Halperin et al. [81] found no differences when comparing different treatment schedules for the same intervention. The majority of the programs had a total duration of four to eight weeks and involved about three weekly sessions, sometimes supplemented by regular practice at home. It can thus be concluded that fine motor trainings in children with ADHD should involve more than one session and the previous training programs reported in this review could serve as a first orientation for devising future interventions. The types of the reviewed interventions were very versatile, comprising offline and online or virtual games, physical activity, specific fine motor components, cognitive training, verbal or automated feedback and multimodal or alternative treatment approaches as effective building blocks.

The positive influence of feedback on motor performance of children with ADHD is consistent with previous research. According to Eliasson et al. [15], children with ADHD seem to rely more on visual feedback while performing goal-directed movements than neurotypical controls. Berninger et al. [89] found visual and verbal cues to support the generation of automated handwriting movements. Feder and Majnemer [61] reported similar results for the effectiveness of instructions in handwriting remediation. Although the reported handwriting interventions were not specifically designed for the ADHD population, Tucha and Lange [49] observed similar effects in children with ADHD, thus suggesting that the previous findings about feedback could be transferred to individuals with the disorder. Rosenbaum et al. [90] pointed at the importance of feedback in the transfer process. When learning a novel perceptual-motor task, it seems to be crucial to receive feedback. While frequent feedback only appears to improve short term performance, infrequent feedback could help the consolidation and transfer of learnt movements to other domains [90].

In general, a playful approach seems to suit the ADHD population, which was reflected in the high reported satisfaction and attendance to the programs. These findings are in line with previous research that emphasizes the need for interest-driven stimulation in ADHD populations [91]. Motivational aspects should be core to every ADHD-specific intervention to improve compliance and performance and this could present a relative strength of novel virtual gaming interventions compared to traditional treatment approaches. One could argue that the ADHD population is specifically prone to the development of comorbid gaming addictions and that videogame interventions could therefore represent a potential risk. On the contrary, the case report by Ruiz-Manrique et al. [84] offers first evidence that the implementation of videogames and apps in the treatment of ADHD could even prevent or remediate video game addiction by providing a clinically approved alternative to common games. Thus, the media affinity of children with ADHD could be used to their advantage in devising motivating serious games for the treatment of fine motor impairments.

### Limitations and implications for research

There are three potential limitations concerning the results of this study. A first limitation is that the screening of articles was conducted by one person only. A second potential limitation concerns the types of articles included in this review. Very few studies were blinded or had an RCT design and a lot of studies were observational, including four case reports. One of these had severe methodological limitations, reporting on alternative medical approaches but lacking scientific proof for the stated evidence. Nevertheless, the implemented treatment seemed to be successful, although the nature of the observed improvements cannot be causally linked to the intervention and could also be attributed to the received attention or to placebo effects rather than the treatment itself. A lot of studies included very subjective measures of motor improvements as parent or teacher reports. Several articles only consisted of a preliminary testing of novel interventions to guide future research. The deductions or conclusions that can be drawn from the included studies are therefore limited. A third limitation concerns the transferability of the observed fine motor improvements. Although some studies included a follow-up, suggesting a lasting effect of the interventions, it remains unclear if the improvements in specific fine motor tasks or handwriting could be transferred to other fine motor domains. The generalization of positive treatment outcomes to behavioral domains and academic achievements reported in several of the included sources still represents a promising observation that could indicate that transfer has occurred.

The present review is a first attempt to address these issues, although the extent of information uncovered is still very limited and shows many gaps. A systematic review does not seem to be applicable to date since there is a lack of experimental studies on the topic. Well-designed RCTs are needed to gain more reliable evidence for the effectiveness of fine motor trainings in children with ADHD.

### Future directions

Despite the methodological weaknesses, the results of this review suggest preliminary evidence for the effectiveness of online games and virtual interventions in the treatment of fine motor impairments in children with ADHD. The *mobile health* *(mHealth)* sector is an emerging field in the context of digitalization with a growing number of serious games being developed for therapeutic purposes [92, 93].

There is a growing body of evidence on the effectiveness of serious games in the rehabilitation of ADHD [93–95]. The implementation of serious games and mHealth apps has a number of reported benefits in providing an accessible and motivating treatment approach [95] with a high ecological validity [96], the ability to collect and report data [97] and to give real-time feedback [98]. For example, the *VirtualClassroom* [96] offers a novel therapeutic tool that involves classic cognitive behavioral therapy in a *virtual reality* (*VR*) environment. Further advantages compared to traditional interventions are the low costs, the often multilingual programs, the safe environment and the possibility to tailor training programs to the specific needs of the individual [92, 94, 99]. According to Wang and Reid [100], VR interventions can either involve *feedback-focused*, *gesture-based* or *haptic-based interactions*. As reported earlier, feedback has proven effective in improving fine motor skills of children with ADHD and this finding could be transferred to the VR domain by implementing feedback-focused interactions that can both provide information and increase motivation during motor learning tasks [101, 102]. Fine motor components could be targeted directly through gesture-based and haptic-based interactions, both including a sensory-motor component.

As seen in several of the reviewed studies, tablets offer new opportunities for the treatment of handwriting and graphomotor impairments in children with ADHD. The availability of tablets in schools is ever increasing. In 2019, more than 8000 tablets were in use in schools in Zurich [103]. For children with ADHD, this is an enormous potential to be better leveraged in the future. Although there already are software tools designed to analyze and aid understanding of the processes underlying handwriting production (i.e. *Ductus* by Guinet et al. or the *ErgoPen* by *Stabilo*), there are only few serious game interventions to support fine and visuo-motor skills in the school setting [104] and even fewer provide immediate feedback to the child or to the teacher (e.g. ErgoPen). Tools like the ErgoPen or other digital pens show that handwriting and digitalization do not necessarily contradict each other and can even be combined. The sensory-motor component of handwriting is crucial for the acquisition of writing and reading skills in schoolchildren and cannot be replaced by typewriting [105]. Handwriting will stay relevant in the digital era although a combination of different media and the use of tablets in the classroom could be a successful approach in combining the advantages of both worlds [105, 106].

## Conclusions

Although fine motor impairments are very common in children with ADHD, they have remained widely undertreated so far. There is a strong need for ADHD-specific, non-pharmacological interventions tailored to the specific characteristics and needs of this population. The present scoping review is a promising step in the investigation of effective treatments of fine motor difficulties in children with ADHD. A variety of training programs and intensities seem to be effective, both in the short and long term. A multimodal approach, verbal or automated feedback and the implementation of motivating serious games appear to be most effective in the treatment of the condition.

Contrary to critical voices, handwriting and fine motor skills will remain an important life skill in the digital era and the latter provides a multitude of opportunities for the treatment of motor comorbidities and for future research in an interdisciplinary field between psychology, occupational therapy and gaming. There is an exciting new world awaiting psychologists outside their laboratories, inviting them to explore the realm of serious gaming for the development of effective training interventions.

## Supplementary Information

**Additional file 1.**

## Data Availability

Not applicable.
