# Supplementary material for: How effective is fine motor training in children with ADHD? A scoping review
Source: BMC Pediatr. 2021 Nov 4;21:490. doi: 10.1186/s12887-021-02916-5 (PMC8567617; doi:10.1186/s12887-021-02916-5)
Supplement: Supplementary file 1 — Additional file 1. [file 12887_2021_2916_MOESM1_ESM.docx]

# Supplementary

## Appendix 1.

*Thesaurus*

ADHD, ADD, attention deficit hyperactivity disorder, attention deficit disorder, fine motor skills, visuo-motor-skills, handwriting, graphomotor activity, graphomotor function training, intervention, program, mHealth, serious game, children, schoolchildren

## Appendix 2.

*Generation of Search String 3*

| AND | **Term 1** | AND | **Term 2** | AND | **Term 3** | NOT | **Term 4** |
| --- | --- | --- | --- | --- | --- | --- | --- |
|  | ADHD |  | “fine motor skills” |  | training* |  | ASD |
| OR | ADD | OR | “visuo motor skills” | OR | intervention* | OR | autism |
| OR | “attention deficit hyperactivity disorder” | OR | handwriting | OR | program* |  |  |
| OR | “attention deficit disorder” | OR | graphomotor* |  |  |  |  |

## Appendix 3.

*Search Strategy*

**PsycINFO**

***Search string 1:* 21 records**(adhd OR add OR “attention deficit hyperactivity disorder” OR “attention deficit disorder”) AND (“fine motor skills” OR “visuo motor skills” OR handwriting OR graphomotor*) AND (training* OR intervention* OR program*)

Limiters: English; Age Groups: School Age (6-12 yrs); Population Group: Human

***Search string 2:* 84 records**
(adhd OR add OR “attention deficit hyperactivity disorder” OR “attention deficit disorder”) AND (“fine motor skills” OR “visuo motor skills” OR handwriting OR graphomotor*)

Limiters: Age Groups: School Age (6-12 yrs)

**Web of Science**

**Search string 3: 112 records**

(adhd OR add OR “attention deficit hyperactivity disorder” OR “attention deficit disorder”) AND (“fine motor skills” OR “visuo motor skills” OR handwriting OR graphomotor*) AND (training* OR intervention* OR program*) NOT (asd OR autism)

**Search string 3: 44 records**

(adhd OR add OR “attention deficit hyperactivity disorder” OR “attention deficit disorder”) AND (“fine motor skills” OR “visuo motor skills” OR handwriting OR graphomotor*) AND (training* OR intervention* OR program*) NOT (asd OR autism)

Refined by: Research areas: Health Care Sciences Services OR Pharmacology Pharmacy OR Psychology OR Neurosciences Neurology OR Rehabilitation OR Education Educational Research OR Pediatrics OR Psychiatry OR Behavioral Sciences

**MEDLINE via PubMed**

**Search string 3: 33 records**

(adhd OR add OR “attention deficit hyperactivity disorder” OR “attention deficit disorder”) AND (“fine motor skills” OR “visuo motor skills” OR handwriting OR graphomotor*) AND (training* OR intervention* OR program*) NOT (asd OR autism)

**Schreibmotorik Institut**

<https://www.schreibmotorik-institut.com/index.php/de/publikationen>

**75 records**

**Amy Lu**

<https://web.northeastern.edu/amylu/publications.html>

**47 records**
